# Hypomethylating Agent-Based Combination Therapies to Treat Post-Hematopoietic Stem Cell Transplant Relapse of Acute Myeloid Leukemia

**DOI:** 10.3389/fonc.2021.810387

**Published:** 2022-01-06

**Authors:** Giulia Ciotti, Giovanni Marconi, Giovanni Martinelli

**Affiliations:** ^1^ Ematologia, Dipartimento di Medicina Traslazionale e di Precisione, Università La Sapienza, Azienda Ospedaliera Policlinico Umberto I, Rome, Italy; ^2^ IRCCS Istituto Romagnolo per lo Studio dei Tumori (IRST) “Dino Amadori”, Meldola, Italy

**Keywords:** relapse, AML—acute myeloid leukemia, allogeneic stem cell transplantation (allo-SCT), hypomethylating agents, azacytidine, DLI, venetoclax, therapy combinations

## Abstract

Allogeneic stem cell transplantation still represents the best curative option for most patients with acute myeloid leukemia, but relapse is still dramatically high. Due to their immunologic activity and safety profile, hypomethylating agents (HMAs) represent an interesting backbone for combination therapies. This review reports mechanism of action, safety, and efficacy data on combination strategies based on HMAs in the setting of post-allogeneic stem cell transplant relapse. Several studies highlighted how HMAs and donor lymphocyte infusion (DLI) combination may be advantageous. The combination strategy of HMA with venetoclax, possibly in association with DLI, is showing excellent results in terms of response rate, including molecular responses. Lenalidomide, despite its well-known high rates of severe graft-versus-host disease in post-transplant settings, is showing an acceptable safety profile in association with HMAs with a competitive response rate. Regarding FLT3 internal tandem duplication (ITD) mutant AML, tyrosine kinase inhibitors and particularly sorafenib have promising results as monotherapy and in combination with HMAs. Conversely, combination strategies with gemtuzumab ozogamicin or immune checkpoint inhibitors did not show competitive response rates and seem to be currently less attractive strategies. Associations with histone deacetylase inhibitors and isocitrate dehydrogenase 1 and 2 (IDH1/2) inhibitors represent new possible strategies that need to be better investigated.

## 1 Introduction

Allogeneic stem cell transplantation (allo-SCT) still represents the most important curative option for most patients with acute myeloid leukemia (AML) eligible for intensive treatment. However, the rate of patients relapsing after an HSCT is dramatically high (1, 2). After relapse, 3-year survival is particularly poor, with less than 20% of patients surviving and not exceeding 4% after early relapse (<6 months) (2–5). Patients who received a reduced-intensity conditioning regimen, who never had graft-versus-host disease (GVHD), who lost donor chimerism, and who have a measurable residual disease (MRD) have a higher risk of relapse (1, 6, 7).

Of note, prophylactic and preemptive strategies are currently adopted in the real-world setting (5); however, this review will focus only on relapse after allo-SCT (post-transplant relapse (PTR)). To date, there is no standard of care for patients who experience PTR. In evidence of molecular recurrence, the first approach is commonly to reduce immunosuppression, administering or not donor lymphocyte infusion (DLI). After PTR, intensive chemotherapy and—whenever feasible—a second allo-SCT from a different source are still the preferred approaches (8). Unfortunately, advancing age, severe comorbidities, frailty, and residual toxicities or organ dysfunction from the previous allo-SCT often exclude the possibility of intensive re-treatment and limit the choice of therapy strategies (2, 9). Of utmost importance, either target mutations may not be present, or target agents may not be clinically available.

**Figure 1:**
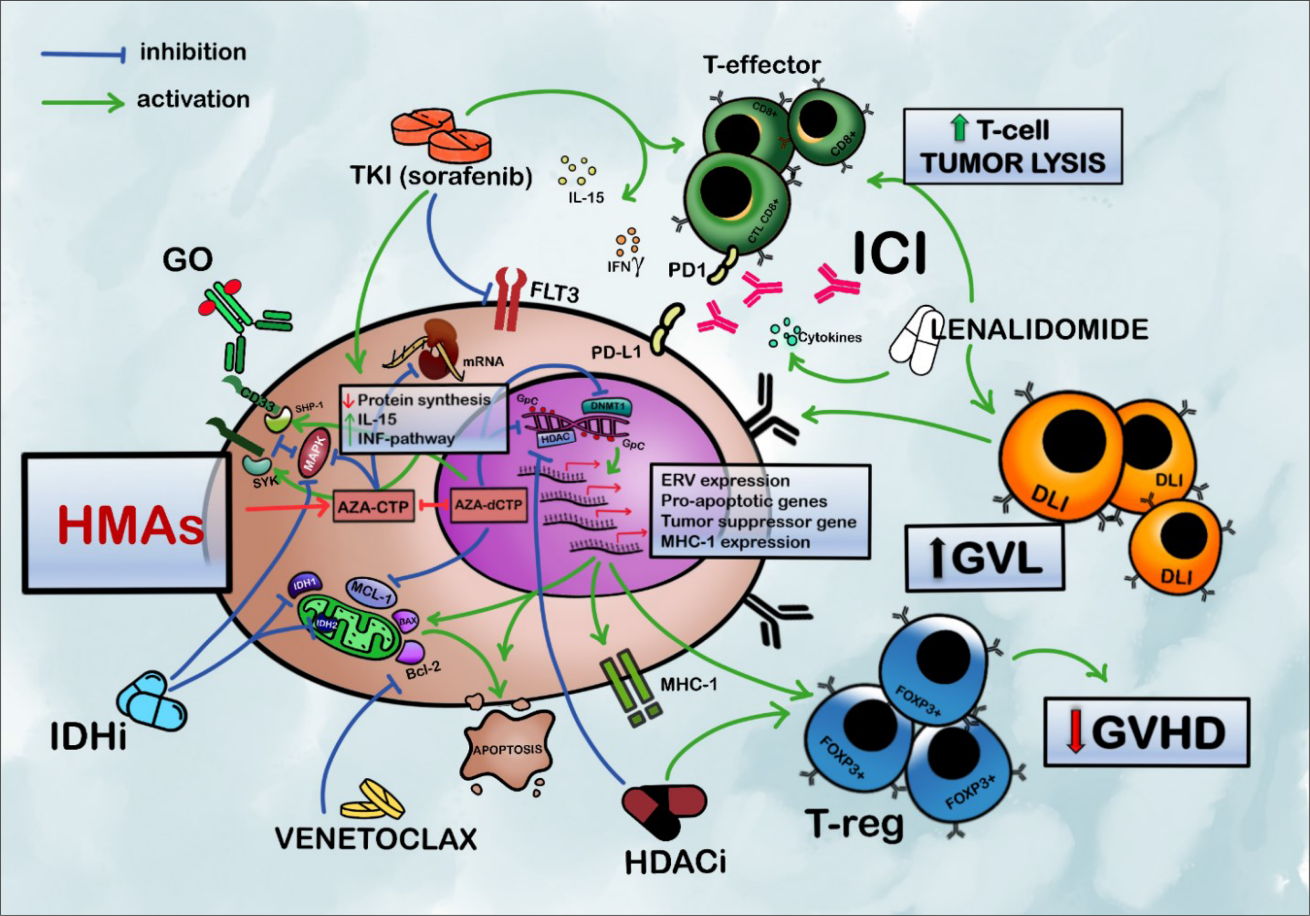
Rationale for HMA-based combination strategies in acute myeloid leukemia relapsing after allogeneic HSCT. HMAs sensitize blasts to the T cell-mediated immune response by upregulation of IFN-pathway genes, increased expression of MHC-1, and ERV. The expansion of FOXP3 + T regs mediated by HMAs facilitates GVL preserving from GVHD. HMAs also upregulate antigen-presenting cells, such as dendritic cells (not pictured). Promoting the modulatory activity of Tregs, AZA greatly reduces the risk of severe GVHD and emphasizes GVL; BCL-2 inhibitors restore mitochondrial apoptotic pathways and sensitize AML cells to HMAs. AZA may, also, synergize to activate BAX pro-apoptotic gene and reduce levels of MCL-1. Sorafenib inhibits FLT3-ITD and the mitogen-activated protein kinase pathway. It also increases cell-mediated immune response enhancing IL-15 production and INFγ-pathway synergizing with allogeneic effective T cells. Lenalidomide increases the activity of T-effectors and the production of pro-inflammatory cytokines; HMAs can reverse the hypermethylation of DNA induced by IDH-mutated clones and synergize with the inhibitory activity of IDHi. HMAs and IDHi, also, synergistically inhibit MAPK/ERK signaling; HDAC inhibitors, especially panobinostat, contribute to the epigenetic modulation and can reinduce the expression of TNF receptors on T-regs favoring control over GVHD and an increase in GVL activity. HMAs increase the expression of PD-1 and PD-L1 representing a possible mechanism of resistance to HMAs. PD-1 inhibition can enhance response to DLI and allogeneic effective T cell and consequent T cell-mediated tumor lysis. HMAs increase CD33 expression with consequently increased uptake of GO by AML cells. It also increases the expression of Syk and SHP1, which contribute to GO-mediated cytotoxicity by inhibiting cell growth. HMAs (mostly AZA) decrease P-glycoprotein expression, which contributes to GO resistance (not pictured). HMAs, hypomethylating agents; IDHi, isocitrate dehydrogenase inhibitors; HDACi, histone deacetylase inhibitors; GO, gemtuzumab ozogamicin; TKI, tyrosine kinase inhibitors; AZA-CTP, azacytidine-cytosine triphosphate; ICI, immune checkpoint inhibitors; DLI, donor lymphocyte infusion; GVL, graft versus leukemia; GVHD, graft-versus-host disease; AZA-dCTP, azacytidine-deoxy cytosine triphosphate; GpC, GpC island; DNMT1, DNA-methyl-transferase 1; HDAC, histone deacetylase; SYK, spleen-associated tyrosine kinase; SHP-1, Src homology region 2 domain-containing phosphatase-1; MAPK, mitogen-activated protein kinase; BCL-2, B-cell leukemia/lymphoma-2; IDH, isocitrate dehydrogenase; FLT3, FMS-like tyrosine kinase 3; MCL-1, myeloid cell leukemia-1; PD1, programmed cell death protein 1; PD-L1, programmed death-ligand 1; T-regs, regulatory T cells; ERV, endogenous retrovirus; MHC-1, major histocompatibility complex, class I.

Hypomethylating agents (HMAs) represent an optimal backbone for combination therapies due to their immunologic activity, their particular mechanisms of action, and their reduced toxicities (10–12). HMAs with or without DLI have frequently been used as a first therapeutic approach for PTR AML, with 2-year overall survival rates between 12% and 29% (13–17), even if most of the available data come from retrospective studies. New combination therapies, particularly with target agents, promise effectiveness that may be even greater than that of chemotherapy (as summarized in **Figure 1**). In this review, we present the rationale and results of the newer HMA-based combinations, summarizing new possible strategies of clinical development in this orphan population.

## 2 HMA-Based Combinations

### 2.1 Hypomethylating Agents Are Optimal Agents to Treat Post-Hematopoietic Stem Cell Transplantation Relapse for Their Toxicity Profile and Mechanism of Action

HMAs were demonstrated to be manageable in patients who received allo-SCT, sparing patients from treatment-related morbidity and mortality. Several studies showed how HMAs, particularly azacytidine (AZA), accelerate reconstitution of T-regulatory lymphocytes in murine models, reducing the risk of severe GVHD (18, 19).

Woo conducted a prospective trial in 39 patients with myelodysplastic syndrome (MDS) or AML (AML = 26) who relapsed within 100 days of allo-SCT; in this group, he documented 3 complete remissions (10%) and 9 partial remissions (30%) (15). De Lima led a study on low-dose AZA post-allo-SCT that demonstrated promising effectiveness (20). In RELAZA and RELAZA2 trials, Platzbecker and colleagues investigated the role of AZA, as a single agent, in a preemptive setting (21, 22). In the RELAZA trial, patients who had chimerism below 80% from peripheral blood after allo-SCT were included; after a median of four cycles of AZA, stable MRD or improvements were obtained in 80% (n = 20) of patients in the absence of hematologic relapse (22). Regarding toxicities, the most common event was grade 3 and 4 neutropenia (80%), mostly reversible with dose reduction; to note, no new onset of GVHD was reported in GVHD-naive patients (15, 20–22). For the aforementioned reasons, combination strategies that are based on HMAs are gaining interest and are widely used in the context of PTR (13, 23, 24). Recently, guadecitabine, a next-generation HMA, has gained interest. It was clinically active with acceptable tolerability (25, 26). It is worth considering guadecitabine in PTR, but randomized clinical pivotal trials after allo-SCT are needed.

### 2.2 Hypomethylating Agents + Donor Lymphocyte Infusion: The Immunological Effects of Hypomethylating Agents Make the Perfect Match With Donor Lymphocyte Infusion

HMAs lead to a switch in the microenvironment and gene expression that may be defined as immune activation response; this may be mediated by various mechanisms including expression of cancer/testis antigens, endogenous retroviruses, upregulation of HLA and costimulatory molecules as treatment-induced non-annotated transcripts, and activations of dendritic cells (27–36). Overall, blasts become more sensitive to the T cell-mediated immune response. AZA also has a direct T-cell effect, expanding the number of CD4+CD25+FOXP3+CD127− regulatory T cells after allo-SCT, which has been shown to facilitate graft versus leukemia (GVL) effect in mice and humans preserving from GVHD (5). Furthermore, HMAs promote antitumor immune signaling by upregulation of IFNγ pathway genes, increased expression of HLA class 1 antigens, and activation of viral defense pathways (19, 37).

As a salvage therapy for PTR, AZA and DLI combination showed meaningful results in several studies. Schröeder conducted a retrospective study on 154 patients of which 124 (81%) were diagnosed with AML. After a median of 4 cycles, the overall response rate (ORR) was 33% with a complete response (CR) rate of 29%; OS at 2 years was 29%. GVHD occurred in less than 30% of patients and was related to DLI (38). In a prospective study, 72 patients have been treated with a schedule of 100 mg of AZA total dose per day on 3 or 5 consecutive days, and DLIs were administered non-homogeneously. CR rate was only 9%, but 44% of the patients presented with temporary disease control; besides, the median survival was 108 days (39). In a retrospective analysis of EBMT, the concurrent administration of DLI did not seem to improve either response rates or OS in patients treated with AZA, and survival rates were determined by time to relapse >6 months (p = 0.001) and marrow % blasts at time of relapse (p = 0.01) (40). An analysis on 28 patients with recurrent AML/MDS after allo-SCT who received AZA-DLI showed that the likelihood of disease response was higher among those patients without an overt hematological relapse, in those who received more cycles of therapy, and in those who had chronic GVHD. Moreover, there was an association between higher CD4+ T cells and prolonged survival (41).

Decitabine (DAC) was also evaluated in association with DLI infusion, mostly in retrospective studies. Sommer et al. described 26 patients treated with 5- or 10-day (when feasible) administration of DAC 20 mg/m^2^. DLIs were given in between DAC cycles; ORR was 19%, and median OS was 4.7 months; GVHD occurred in 4 patients (including 1 GVHD-related death). The majority of patients have stable disease control with a median duration of 101 days (42). In another study, 36 patients received 1 to 11 courses of DAC (median 2). DAC was the first salvage therapy in 44% of patients, whereas 56% had previously received ≥1 salvage therapy, including AZA. ORR was 25% with a CR rate of 17%, and 2-year OS rate was 11% without any difference between first salvage and pretreated patients. Notably, DAC can induce durable remissions even after AZA failure, even if in a different setting (14). The rate of acute GVHD was 19% (chronic 5%), which appears to be favorable in comparison with historic controls using DLI alone (16).

The dose and timing of DLI in combination with AZA and combinations with third agents remain to be explored. Also, the benefits of prophylactic versus preemptive versus at relapse treatment have to be defined.

### 2.3 Azacytidine + Venetoclax +/− Donor Lymphocyte Infusion: The BCL-2 Inhibitor Venetoclax Is Entering the Game After Outstanding First-Line Results

BCL-2 inhibitors release the power of pro-apoptotic proteins and sensitize AML cells to HMAs (43). The combination of venetoclax (VEN) and HMAs has shown promising efficacy in elderly patients with AML (44, 45). Overall, the addition of VEN seems to diminish the tolerance, especially in terms of hematological toxicities (44, 45).

In a recent study in relapsed/refractory (R/R) AML, the treatment of VEN with other therapies (HMAs in 72% of cases) guaranteed an objective response of 21% and a median OS of 3.0 months (range: 0.5–8.0 months) on a group of 43 patients including patients with prior allo-SCT; 77% of patients had previously failed to respond to other HMA-based therapies. The most relevant adverse events were prolonged cytopenia (most of them were present before treatment initiation and related to AML), which often led to grade ≥3 febrile neutropenia and hospitalizations (46). A retrospective study conducted in 11 centers finds that in R/R AML post-intensive chemotherapy, treatment with VEN combined with HMAs leads to 76% neutrophil recovery and 59% platelet count recovery in patients who survive for over two cycles of treatments; of note, 2% of them relapsed after a prior allo-SCT (47). In another retrospective study on 33 R/R AML patients, of whom 13 underwent a previous allo-SCT, ORR was 64% with a 2-years OS of 53%. Also in this cohort, almost 60% of patients had failed to respond to HMA therapy (48).

Efficacy of VEN+AZA and DLI for patients with PTR AML has few data; recently, Zhao et al. evaluated the efficacy and tolerability of the association between VEN, AZA, and DLI in a prospective study on 26 patients. This combination achieved a composite CR rate of 26.9%; in terms of survival, the median event-free survival (EFS) was 120 days, and the median OS was 285 days. As for tolerance, the most common adverse events were agranulocytosis, anemia, and thrombocytopenia, with most of them being grade III/IV. No serious adverse events were reported (6). Overall, VEN+AZA in PTR may deserve an extensive review of safety; the combination with DLI may require the analysis of data on a higher number of patients.

### 2.4 Hypomethylating Agents + Tyrosine Kinase Inhibitors to Drug FLT3

The proliferative potential and unfavorable prognoses associated with FLT3 mutations also persist after allo-SCT. Single-agent gilteritinib is the standard of care for FLT3 positive AML in the first relapse; however, most of the patients with PTR already failed to respond to the compound (49). The off-label combination of gilteritinib with HMAs is used in the real-life setting; however, it is poorly reported in the literature (50).

Sorafenib is an orally active multi-kinase inhibitor with potent activity against FLT3 internal tandem duplications (ITDs) and the Raf/ERK/mitogen-activated protein kinase pathway (51). Moreover, sorafenib elicits a cell-mediated immune response, increasing IL-15 production by FLT3-ITD AML cells and synergizing the allogeneic CD8+ T cell. Therefore, sorafenib might contribute to an immune-mediated cure of relapsed FLT3-ITD mutated AML especially in PTR (52). In the phase II study, 37 R/R FLT3 ITD mutant AML patients (7 with a history of allo-SCT) received AZA and sorafenib 400 mg BID. ORR was 46% with 6 (16%) CR and 10 (27%) CR without complete reconstitution. The median duration of CR was 2.3 months (11). In a retrospective study focusing on PTR, 8 patients received a median of 5 AZA and sorafenib cycles, with 4 of them together with DLI. Four patients (50%) achieved CR, and 3 were negative for MRD; of note, 2 patients remained in remission after discontinuation of sorafenib. Acute GVHD was reported in 4 patients and chronic not-extensive GVHD in 2. The median OS was 322 days (53).

The combination of midostaurin and AZA, in a first-line setting, was shown to be poorly tolerable and with a non-brilliant CR rate when compared with the VEN/AZA combination (54). In a phase I study, this combination was also evaluated in FLT3 wild-type R/R patients and was found to be safe and tolerable, but response rates were comparable with those of AZA alone (55). A study on the combination of quizartinib with AZA or low-dose cytarabine for the treatment of patients with R/R AML is ongoing and still recruiting (NCT01892371).

Maiti and colleagues reported triplet therapy with VEN, FLT3 inhibitor, and DAC for 13 R/R FLT3-mutated AML (4 with PTR). In 8 patients with R/R AML and prior exposure to an FLT3i, the CR rate was 63%, with FLT3 negativity by pathological (CR) PCR in 4/4 (50).

Patients with PTR, previously exposed to tyrosine kinase inhibitor (TKI), could harbor FLT3+ RAS-mutant clones and display resistance to FLT3 inhibitor as a single agent (56). The combination of HMAs and TKI and possibly other target molecules could overcome some resistance mechanisms. Prospective studies are needed to define the safety and efficacy of different FLT3 inhibitors in combination therapy, especially in PTR settings.

### 2.5 Hypomethylating Agents + Lenalidomide Has High Antileukemic Activity With a High Price: Finding a Safe Dose Required a Dose-Escalation Study

Lenalidomide (LEN) demonstrates antileukemic activity in patients with PTR; however, it was historically associated with high rates of severe or life-threatening GVHD and was usually contraindicated post-allo-SCT (6, 57). Craddock et al. conducted a dose-finding study of LEN administered in combination with AZA in patients with PTR of AML (n = 24) and MDS (n = 5). The maximum tolerated dose of LEN for the combination with AZA was 25 mg. In the entire patient set, ORR was 24% with a median duration of remission of 11 months and median OS of 27 months for responders. The median OS of the responders was better than 10 months’ median OS observed in non-responders (p = 0.004). Three patients developed grade II/IV GVHD, without any GVHD-related mortality. Interesting to note, combined AZA+LEN therapy does not affect the immune setting in terms of reversal of T-cell phenotype, and T-cell phenotype does not correlate with response to AZA+LEN (58). After this seminary trial, the AZA+LEN combination deserves to be explored extensively in this setting. Investigators from Dusseldorf completed a prospective single-arm phase II trial based on AZA+LEN (5 mg/die × 21 days) + DLI for secondary AML, MDS, and chronic myelomonocytic leukemia (CMML) relapsing after allo-SCT, and results are expected.

### 2.6 Hypomethylating Agents + Isocitrate Dehydrogenase 1 and 2 Inhibitors Promise to Be a Good Compromise Between Toxicity and Effectiveness in the Future

Mutations in isocitrate dehydrogenase 1 and 2 (IDH1/2) can be found in approximately 20% of AML cases (59). Ivosidenib and enasidenib are oral, targeted, small-molecule inhibitors of mutant IDH1 and mutant IDH2, respectively. These molecules are approved by the Food and Drug Administration (FDA), as monotherapy, for the treatment of R/R AML with IDH1/2 mutation (60, 61). Despite the mechanism of action of these compounds suggesting a synergistic action with HMAs, only a few prospective studies have been conducted. From a biological point of view, at least IDH1 inhibition synergizes with AZA, depleting leukemia stem cells *via* inactivation of MAPK/ERK and RB/E2F signaling (6). In a frontline setting, ivosidenib in association with AZA obtained 78.3% (18/23 patients) and a CR rate of 61% (14/23 patients), with a median follow-up of 16 months; the median duration of response in responders had not been reached. This combination was well tolerated, with no dose-limiting toxicities and with a safety profile consistent with that of ivosidenib and AZA monotherapies (62). An ongoing study, still in the recruitment phase, is evaluating the efficacy of enasidenib and AZA association in patients with R/R AML with IDH2 mutation (NCT03683433).

### 2.7 Hypomethylating Agents + Histone Deacetylase Inhibitors Are a Possible Frontier of Development

Preclinical and clinical studies have shown that combinations of AZA and histone deacetylase inhibitor (HDACi), especially panobinostat (a non-selective HDACi), can reinduce the expression of TNF receptor-2 (TNFR2)-expressing Tregs, reduce pro-inflammatory cytokine, act on dendritic cells, and exert direct antitumor activity (63–67). Vorinostat was also effective in GVHD prevention (68). The combination has synergistic effects against myeloid blasts and seems to be tolerable. The combination was also proved to be active in first-line AML and MDS in phase II studies (69, 70). PANOBEST trial was a phase I/II study designed to assess the efficacy and feasibility of single-agent panobinostat in high-risk MDS/AML patients who underwent allo-SCT. With a median follow-up of 22 months, the probabilities of 2 years’ OS and RFS were 81% and 75%, which were better than previous outcomes (71). A feasibility study on preemptive panobinostat and DAC followed by DLI was conducted with good tolerability and effectiveness results (68). Despite the possible rationale for use, studies evaluating the efficacy and safety of this combination in PTR are still needed; NCT04326764 is testing the hypothesis that panobinostat could be effective in the prevention of relapse also conferring good GVHD and safety profile.

### 2.8 Hypomethylating Agents + Immune Checkpoint Inhibitors +/− Donor Lymphocyte Infusion: A Fascinating Biological Rationale That Is Not Holding the Promises

Other than immunological changes that were mentioned in Section 2.1, HMAs increase the expression of PD-1 and PD-L1, especially in AML (72, 73). Some authors have assumed that upregulation of the immune checkpoint inhibitor (ICI) signaling may be a mechanism of resistance to HMAs. Moreover, PD1 expression on T cells significantly increase in AML patients who had PTR (74). PD-1/PD-L1 inhibition can enhance donor cytotoxic T-cell responses and may promote GVL; however, there is evidence that also GVHD is facilitated (75–77).

The efficacy of ICIs for hematologic malignancies has generally not been impressive, and studies in AML have shown acceptable safety profile but generally modest activity (78) with a slightly augmented activity with PD-L1 inhibitors (79). The combination of the PD1 inhibitor nivolumab with AZA was studied in 70 patients with R/R AML of whom 13 had a previous HSCT. The combination yielded an ORR of 33% (CR rate of 22%). In multivariate analysis, no prior HMAs increased pre-therapy BM CD3+ T cells, and the presence of ASXL1 mutation had a trend of improving ORR. Patients in advanced salvage have depleted CD3+, CD8+, and CD4+Teff T cells and may be less likely to benefit from T cell-dependent therapies (80). The safety and efficacy of AZA and avelumab (a PD-L1 inhibitor) combination were explored in a phase Ib/II clinical trial in patients with R/R AML. Although the combination was well tolerated, the study was terminated early because clinical activity was limited. The ORR was only 11%, with a median OS of 4.8 months (81).

Some anecdotal cases have shown how the possible association between AZA, PD1 inhibitors, and DLI can represent an effective and safe strategy; PD1 inhibitors combined with HMAs and DLI may enhance the GVL activity of the hosts (donor chimerism) and infused donors’ lymphocytes (82). Second-generation ICI, targeting TIM or LAG3, or SIRP1a/CD47 directed therapies may give new life to this strategy.

### 2.9 Hypomethylating Agents + Gemtuzumab Ozogamicin: Toxicities Limited Further Developments

Gemtuzumab ozogamicin (GO) is an anti-CD33 immunoconjugate, approved for combination with intensive chemotherapy for first-line AML (83, 84). HMA treatment increases CD33 expression and reduces the expression of P-glycoprotein, which mediates resistance to GO (85). Several pieces of evidence suggested that treatment with HMAs may enhance the effectiveness of GO against AML blasts (86). A phase I/II study showed that using AZA and GO for R/R AML patients gave significant hematologic toxicity and related severe clinical sequelae (87). GO usage remains tricky in this setting, and the safe dose remains to be completely explored when combined with HMA therapy.

## 3 Discussion

Nowadays, for most patients, HMA-based therapies may be an optimal strategy in the setting of AML recurrence after allo-SCT. No direct comparison with intensive therapies was performed, and no precise fitness was defined; however, clinical judgment often assigns patient to non-intensive salvages in this setting. In this review, we summarized optimal strategies that are based on HMAs; **Table 1** contains the results of meaningful experiences in this setting.

**Table 1:** Studies on hypomethylating agent-based combination therapy in AML relapsed post-HSCT (including only studies in which more than 10 patients with post-allogeneic stem cell transplant relapse were considered).

	Author	Description	ORR	CR/CRi	OS (month or %)	FU	AE grade III/IV
AZA + DLI	Claiborne J et al. (41)	Retrospective		36%	9 months	40 months	cGVHD (20%)
		28 (AML = 14)					Infection (46%)
		Age: 57 (22–69)					
DAC + DLI	Schröeder T et al. (16)	Retrospective	25 %	17%	11% at 2 years	5 months	Myelosuppressi on (47%)
		36					cGVHD (5%)
		Age: 56 (21–72)					
AZA +/− DLI	Craddock C et al. (40)	Retrospective	29.3%	15.3%	12% at 2 years	2 years	Infection/sepsis
		181 pt (AML = 116)			48% in CR pt		
AZA + DLI	Schröeder T et al. (38)	Retrospective	33%	27%	29% at 2 years	13 months	aGVHD (35%)
		154 pt (AML = 124)					cGVHD (27%)
		Age: 55 (21–72)					
AZA + DLI	Steinmann J et al. (39)	Retrospective	55%	9%	3.6 months		Febrile neutropenia
		72 pt (AML = 62)					Infection/sepsis
		Age: 62 (20–75)					
DAC + DLI	Sommer S et al. (42)	Retrospective	19%	15%	4.7 months		Neutropenia
		26 pt (AML = 18)					Infection
		Age: 59 (21–84)					
AZA + VEN	Ganzel C et al. (47)	Retrospective		52%	4.5 months	5.5 months	Myelosuppression
		40 pt (17 post-HSCT)					Febrile neutropenia infections
		Age: 67 (21–82)					
HMA + VEN	Aldoss I et al. (48)	Retrospective	64%	30%	53% at 1 year	6.5 months	Myelosuppression
		33 pt (13 post-HSCT)					Febrile neutropenia
		Age: 62 (19–81)					Sepsis
AZA + VEN + DLI	Zhao P et al. (6)	Prospective	62%	27%	9.5 months	30 months	Myelosuppression (100%)
		26 pt					Fever (57%)
		Age: 35 (16–60)					
HMA + VEN +/− DLI	Schuler E et al. (88)	Retrospective	47%	33%	3.7 months	8.4 months	Myelosuppression (81%)
		32 pt					Fever
		Age: 54 (31–72)					Infection (72%)
AZA + NIVO	Daver N et al. (80)	Prospective	33%	33%	6.3 months	21.4 months	
		70 (13 post-HSCT)			16.2 months in CR pt		
		Age: 70 (22–90)					
AZA + LENA	Craddok C et al. (58)	Phase Ib	24%	40%	10 months	23 months	Febrile neutropenia
		29 (AML = 24)			27 months in responders		Sepsis
		Age: 54 (18–73)					
AZA + GO	Mendeiros BC et al. (87)	Phase I/II		18%			Myelosuppression
		50 pt (17 post-HSCT)					Febrile neutropenia (76%)
		Age: 64 (29–82)					Sepsis (38%)

ORR, overall response rate; CR, complete remission; CRi, complete remission with incomplete count recovery; OS, overall survival; FU, median follow-up; AE, adverse events; HMA, hypomethylating agents; AZA, azacytidine; DLI, donor lymphocyte infusion; DAC, decitabine; VEN, venetoclax; NIVO, nivolumab; LENA, lenalidomide; GO, gemtuzumab ozogamicin; cGVHD, chronic graft-versus-host disease; aGVHD, acute graft-versus-host disease.

Whereas the results from HMAs and HMAs in combination with DLI are well known, experiences on newer combinations are limited and mostly non-exclusively performed in PTR. VEN- and LEN-based combinations may be considered the most interesting strategies at the moment, together with sorafenib in the subset with FLT3 ITD mutant AML who already failed to respond to gilteritinib. Particularly, LEN combination toxicity seems to be not related to the dose and is also acceptable. VEN may be hematotoxic, and fine-tuning of the dosage and time of exposure may be required in the future, especially in early PTR. Associations of HMAs with HDACi and IDH1/2 inhibitors represent new possible strategies that are being explored.

PTR should be better explored *per se*, rather than in the conundrum of the entire relapse setting. It is our opinion that allo-SCT provides an immunological platform of which we have to harness the power (not limiting to T cells). The use of novel strategies in the setting of minimal recurrence of the disease may be more effective (14, 16, 41). A particular frailty profile that has to be understood requires a fine-tuning of dose and exposure to selected (mostly myelotoxic or lymphocyte activating) drugs. Dedicated trials in the PTR will be warranted, and dose escalation will be re-applied whenever safety will have to be discussed for the mechanisms of action, with particular regard to hematological toxicities and GVHD.

## Author Contributions

GC performed the literature review and collected safety and effectiveness outcomes. All the authors contributed to the writing and review and approved the manuscript.

## Conflict of Interest

GMarc: consultant/speaker bureau of Menarini/stemline, Pfizer, and Astellas and research support from Pfizer, AbbVie, and AstraZeneca. GMart declares the following conflict of interests: consultant/advisor/speaker bureau of Ariad/Incyte, Pfizer, Celgene/BMS, Amgen, Roche, AbbVie, GlaxoSmithKline, Astellas, Daiichi Sankyo, Takeda, and Janssen and research support from Pfizer, AbbVie, AstraZeneca, Daiichi Sankyo, Takeda, and Ariad/Incyte.

The remaining author declares that the research was conducted in the absence of any commercial or financial relationships that could be construed as a potential conflict of interest.

## Publisher’s Note

All claims expressed in this article are solely those of the authors and do not necessarily represent those of their affiliated organizations, or those of the publisher, the editors and the reviewers. Any product that may be evaluated in this article, or claim that may be made by its manufacturer, is not guaranteed or endorsed by the publisher.
